# Supplementary material for: Unveiling the Bioactive Potential of the Invasive Jellyfish Phyllorhiza punctata Through Integrative Transcriptomic and Proteomic Analyses
Source: Biomolecules. 2025 Aug 4;15(8):1121. doi: 10.3390/biom15081121 (PMC12383608; doi:10.3390/biom15081121)
Supplement: Supplementary file 1 [file biomolecules-15-01121-s001.zip › Suplementary Material/FileS3/PEPFOLD4/XP_065060802_4203/clusters.html]

 PEP-FOLD clusters


Download archive of all models  here   

|  |  |  |
| --- | --- | --- |
| # | file | sOPEP |
| # cluster 1 - Size: 24 |  |  |
|  | XP\_065060802\_4203-model1 | -24.9927 |
|  | XP\_065060802\_4203-model1.1 | -24.9366 |
|  | XP\_065060802\_4203-model1.2 | -24.8883 |
|  | XP\_065060802\_4203-model1.3 | -24.8811 |
|  | XP\_065060802\_4203-model1.4 | -24.8767 |
|  | XP\_065060802\_4203-model1.5 | -24.858 |
|  | XP\_065060802\_4203-model1.6 | -24.8234 |
|  | XP\_065060802\_4203-model1.7 | -24.8047 |
|  | XP\_065060802\_4203-model1.8 | -24.7663 |
|  | XP\_065060802\_4203-model1.9 | -24.7309 |
|  | XP\_065060802\_4203-model1.10 | -24.6937 |
|  | XP\_065060802\_4203-model1.11 | -24.6858 |
|  | XP\_065060802\_4203-model1.12 | -24.6587 |
|  | XP\_065060802\_4203-model1.13 | -24.6493 |
|  | XP\_065060802\_4203-model1.14 | -24.6493 |
|  | XP\_065060802\_4203-model1.15 | -24.6487 |
|  | XP\_065060802\_4203-model1.16 | -24.6462 |
|  | XP\_065060802\_4203-model1.17 | -24.6158 |
|  | XP\_065060802\_4203-model1.18 | -24.586 |
|  | XP\_065060802\_4203-model1.19 | -24.5793 |
|  | XP\_065060802\_4203-model1.20 | -24.5712 |
|  | XP\_065060802\_4203-model1.21 | -24.5531 |
|  | XP\_065060802\_4203-model1.22 | -24.5016 |
|  | XP\_065060802\_4203-model1.23 | -24.4822 |
| # cluster 2 - Size: 17 |  |  |
|  | XP\_065060802\_4203-model2 | -24.9825 |
|  | XP\_065060802\_4203-model2.1 | -24.9164 |
|  | XP\_065060802\_4203-model2.2 | -24.8981 |
|  | XP\_065060802\_4203-model2.3 | -24.8388 |
|  | XP\_065060802\_4203-model2.4 | -24.8143 |
|  | XP\_065060802\_4203-model2.5 | -24.7951 |
|  | XP\_065060802\_4203-model2.6 | -24.7425 |
|  | XP\_065060802\_4203-model2.7 | -24.7245 |
|  | XP\_065060802\_4203-model2.8 | -24.7052 |
|  | XP\_065060802\_4203-model2.9 | -24.7052 |
|  | XP\_065060802\_4203-model2.10 | -24.7041 |
|  | XP\_065060802\_4203-model2.11 | -24.6465 |
|  | XP\_065060802\_4203-model2.12 | -24.6378 |
|  | XP\_065060802\_4203-model2.13 | -24.6137 |
|  | XP\_065060802\_4203-model2.14 | -24.5988 |
|  | XP\_065060802\_4203-model2.15 | -24.5974 |
|  | XP\_065060802\_4203-model2.16 | -24.5782 |
| # cluster 3 - Size: 6 |  |  |
|  | XP\_065060802\_4203-model3 | -24.9381 |
|  | XP\_065060802\_4203-model3.1 | -24.859 |
|  | XP\_065060802\_4203-model3.2 | -24.8532 |
|  | XP\_065060802\_4203-model3.3 | -24.8356 |
|  | XP\_065060802\_4203-model3.4 | -24.691 |
|  | XP\_065060802\_4203-model3.5 | -24.6818 |
| # cluster 4 - Size: 10 |  |  |
|  | XP\_065060802\_4203-model4 | -24.923 |
|  | XP\_065060802\_4203-model4.1 | -24.8928 |
|  | XP\_065060802\_4203-model4.2 | -24.8698 |
|  | XP\_065060802\_4203-model4.3 | -24.8157 |
|  | XP\_065060802\_4203-model4.4 | -24.7949 |
|  | XP\_065060802\_4203-model4.5 | -24.7843 |
|  | XP\_065060802\_4203-model4.6 | -24.6775 |
|  | XP\_065060802\_4203-model4.7 | -24.6683 |
|  | XP\_065060802\_4203-model4.8 | -24.5521 |
|  | XP\_065060802\_4203-model4.9 | -24.5283 |
| # cluster 5 - Size: 20 |  |  |
|  | XP\_065060802\_4203-model5 | -24.9177 |
|  | XP\_065060802\_4203-model5.1 | -24.8854 |
|  | XP\_065060802\_4203-model5.2 | -24.8605 |
|  | XP\_065060802\_4203-model5.3 | -24.8599 |
|  | XP\_065060802\_4203-model5.4 | -24.8193 |
|  | XP\_065060802\_4203-model5.5 | -24.8079 |
|  | XP\_065060802\_4203-model5.6 | -24.7998 |
|  | XP\_065060802\_4203-model5.7 | -24.7811 |
|  | XP\_065060802\_4203-model5.8 | -24.7793 |
|  | XP\_065060802\_4203-model5.9 | -24.7144 |
|  | XP\_065060802\_4203-model5.10 | -24.7144 |
|  | XP\_065060802\_4203-model5.11 | -24.6953 |
|  | XP\_065060802\_4203-model5.12 | -24.694 |
|  | XP\_065060802\_4203-model5.13 | -24.6821 |
|  | XP\_065060802\_4203-model5.14 | -24.6578 |
|  | XP\_065060802\_4203-model5.15 | -24.6578 |
|  | XP\_065060802\_4203-model5.16 | -24.6543 |
|  | XP\_065060802\_4203-model5.17 | -24.616 |
|  | XP\_065060802\_4203-model5.18 | -24.616 |
|  | XP\_065060802\_4203-model5.19 | -24.5944 |
| # cluster 6 - Size: 17 |  |  |
|  | XP\_065060802\_4203-model6 | -24.8399 |
|  | XP\_065060802\_4203-model6.1 | -24.7942 |
|  | XP\_065060802\_4203-model6.2 | -24.7846 |
|  | XP\_065060802\_4203-model6.3 | -24.7846 |
|  | XP\_065060802\_4203-model6.4 | -24.7761 |
|  | XP\_065060802\_4203-model6.5 | -24.7761 |
|  | XP\_065060802\_4203-model6.6 | -24.7731 |
|  | XP\_065060802\_4203-model6.7 | -24.6853 |
|  | XP\_065060802\_4203-model6.8 | -24.6853 |
|  | XP\_065060802\_4203-model6.9 | -24.6704 |
|  | XP\_065060802\_4203-model6.10 | -24.6412 |
|  | XP\_065060802\_4203-model6.11 | -24.6412 |
|  | XP\_065060802\_4203-model6.12 | -24.6383 |
|  | XP\_065060802\_4203-model6.13 | -24.6383 |
|  | XP\_065060802\_4203-model6.14 | -24.6367 |
|  | XP\_065060802\_4203-model6.15 | -24.6217 |
|  | XP\_065060802\_4203-model6.16 | -24.584 |
| # cluster 7 - Size: 6 |  |  |
|  | XP\_065060802\_4203-model7 | -24.6254 |
|  | XP\_065060802\_4203-model7.1 | -24.6142 |
|  | XP\_065060802\_4203-model7.2 | -24.5723 |
|  | XP\_065060802\_4203-model7.3 | -24.5723 |
|  | XP\_065060802\_4203-model7.4 | -24.5723 |
|  | XP\_065060802\_4203-model7.5 | -24.5723 |
|  |  |  |
